# Lifestyle Score and Genetic Factors With Hypertension and Blood Pressure Among Adults in Rural China

**DOI:** 10.3389/fpubh.2021.687174

**Published:** 2021-08-17

**Authors:** Miaomiao Niu, Liying Zhang, Yikang Wang, Runqi Tu, Xiaotian Liu, Chongjian Wang, Ronghai Bie

**Affiliations:** ^1^Department of Epidemiology and Biostatistics, College of Public Health, Zhengzhou University, Zhengzhou, China; ^2^School of Information Engineering, Zhengzhou University, Zhengzhou, China; ^3^Department of Preventive Medicine, Henan University of Chinese Medicine, Zhengzhou, China

**Keywords:** blood pressure, hypertension, lifestyle score, genetic risk score, joint effect

## Abstract

**Background:** Although high genetic risk and unhealthful lifestyles are associated with a high risk of hypertension, but the combined relationship between lifestyle score and genetic factors on blood pressure remains limited, especially in resource-constrained areas.

**Aim:** To explore the separate and joint effects between genetic and lifestyle factors on blood pressure and hypertension in rural areas.

**Methods:** In 4,592 adults from rural China with a 3-year of follow-up, a genetic risk score (GRS) was established using 13 single nucleotide polymorphisms (SNPs) and the lifestyle score was calculated including factors diet, body mass index (BMI), smoking status, drinking status, and physical activity. The associations of genetic and lifestyle factors with blood pressure and hypertension were determined with generalized linear and logistic regression models, respectively.

**Results:** The high-risk GRS was found to be associated with evaluated blood pressure and hypertension and the healthful lifestyle with diastolic blood pressure (DBP) level. Individuals with unhealthful lifestyles in the high GRS risk group had an odds ratio (OR) (95% CI) of 1.904 (1.006, 3.603) for hypertension than those with a healthful lifestyle in the low GRS risk group. Besides, the relative risk (RR), attributable risk (AR), and population attributable risk (PAR) for unhealthful lifestyle are 1.39, 5.87, 0.04%, respectively, and the prevented fraction for the population (PFP) for healthful lifestyle is 9.47%.

**Conclusion:** These results propose a joint effect between genetic and lifestyle factors on blood pressure and hypertension. The findings provide support for adherence to a healthful lifestyle in hypertension precision prevention.

**Clinical Trial Registration:** The Henan Rural Cohort Study has been registered at the Chinese Clinical Trial Register (Registration number: ChiCTR-OOC-15006699). http://www.chictr.org.cn/showproj.aspx?proj=11375.

## Introduction

Hypertension is reported to be the major contributor to premature death and cardiovascular disease (CVD) worldwide (1, 2). The prevalence of hypertension was 31.1% in 2010, 28.5% in high-income countries, and 31.5% in low- and middle-income countries (3). An estimated 8.5 million deaths in 2015 were due to systolic blood pressure (SBP) >115 mm Hg, 88% of these deaths were in low- and middle-income countries (4). Worse still, the prevalence of hypertension in low- and middle-income countries is still on the rise (5). In China, nearly half of the Chinese adults aged 35–75 year had hypertension, but only 7.2% of them are under control (6). The widening disparity in the prevalence of hypertension indicates that high blood pressure remains a global public health problem that needs to be addressed urgently, especially in low- and middle-income countries (5).

Studies have shown that increasing age can lead to higher blood pressure levels (7). Healthful lifestyle factors such as a healthful diet, increased exercise, and reduced alcohol use are also important contributors to lower blood pressure levels (8–11). In addition, large genome-wide association studies (GWAS) studies have identified a large number of single nucleotide polymorphisms (SNPs) associated with blood pressure globally (12–15). Reports suggest that the associations between genetic factors and hypertension are more easily identified when individual loci are integrated into genetic risk scores (GRS) (16, 17).

A study in the United States suggested that premature death decreased and life expectancy increased in populations with higher lifestyle scores, which included five lifestyle factors, namely, smoking, body mass index (BMI), physical activity, alcohol intake, and diet quality score (18). In addition, a study in China showed that a healthy lifestyle was associated with lower type 2 diabetes, regardless of genetic factors (19). Some studies have confirmed the independent role of GRS and lifestyle scores in different diseases (20–22). However, few studies have focused on hypertension and blood pressure levels, especially in resource-limited areas. Therefore, the purpose of this study was to explore the separate and joint effects between GRS and lifestyle scores on hypertension and to provide a basis for better understanding the impact of the healthful lifestyle on the prevention of hypertension.

## Materials and Methods

### Study Population

The Henan Rural Cohort, which was launched in Henan Province, China, is a study that examines risk factors for non-communicable diseases in rural China (23). Subjects in the current study were from a subset of The Henan Rural Cohort dedicated to the study of genetic factors, with a total of 8,268 subjects, who have completed a 3-year follow-up survey. These populations were surveyed at baseline in 2015 and a follow-up survey was completed in 2018. In detail, those with hypertension at baseline, those with incomplete genetic information, and those without essential information were excluded. A flow chart was provided in Supplementary Figure 1 to briefly illustrate the source and exclusions of the study population. The study was finally conducted among 4,592 subjects. All the subjects signed written informed consent. This study was approved by the Zhengzhou University Life Science Ethics Committee.

### Lifestyle Factors and Covariates

Each subject underwent a face-to-face questionnaire during the baseline and follow-up surveys, which was administered by well-trained investigators. The questionnaire contained a series of questions, which include general demographic information such as age, sex, educational level, income, marital status, information on diet, lifestyle information such as smoking, alcohol consumption, and physical activity, and personal and family history of the disease. The English version of the questionnaire has been reported in a previous article (24). Educational level was divided into three categories: Primary and below, Junior, and Senior and above. Income was divided into three categories according to per capita monthly income: <1,000 RMB, 1,000–2,999 RMB, and more than 3,000 RMB.

The anthropometric indices, such as height and weight were measured at least twice for each subject, and the average of these two measurements was used as the value of the corresponding index. BMI is an internationally recognized measure of the degree of obesity of an individual, which was expressed as height (meter) divided by the square of body weight (kilogram).

### Blood Pressure Measurements and Hypertension

The outcomes of this study were blood pressure levels after 3 year and hypertension diagnosis in the past 3 year. Blood pressure values were measured after the subjects had been sitting still for 5 min. The blood pressure measurements were repeated three times and the average value was taken as the blood pressure value of the subject.

According to the Chinese guidelines for the management of hypertension (25), hypertension is defined as SBP greater than or equal to 140 mmHg or diastolic blood pressure (DBP) greater than or equal to 90 mmHg. Furthermore, subjects who took antihypertensive medication within 2 week were considered hypertensive patients.

### Lifestyle Score

The lifestyle score consists of five lifestyle factors: diet, BMI, smoking status, physical activity, and drinking status, each of which is divided into two categories: healthful and unhealthful. Each unhealthful lifestyle factor has a lifestyle score of 0 and a healthful lifestyle of 1. The detailed definitions for the healthful and unhealthful lifestyle factors are described below and in Supplementary Table 2.

Diet quality was assessed according to the Chinese Healthy Eating Index (CHEI) (26). Each of the nine foods in the CHEI (whole grains, fish, eggs, dairy food, vegetables, fruits, been products, nut, and red meat) was assigned a score from 4 to 0 (except for red meat, which was assigned a score from 0 to 4) in order of five frequencies (daily, weekly, monthly, annually, and never). The range of CHEI was 3–28, with the top 40% (≥ 17) of the highest CHEI defined as the healthful diet and the bottom 60% (<17) of the low CHEI defined as the unhealthful diet (27).

BMI between 18.5 (inclusive) and 23.9 (inclusive) is defined as the healthful BMI, while a BMI <18.5 and >24 is defined as the unhealthful BMI (28).

Based on the smoking status and years of cessation, healthful smoking status was defined as never smoking or having quit smoking for more than 30 years (inclusive), and unhealthful smoking status was defined as current smoking or having quit smoking for <30 years (27).

The physical activity of each individual was divided into three groups of low, moderate, or high, according to the International Physical Activity Questionnaire (IPAQ) recommended by WHO (29). Moderate and high physical activity is defined as healthful physical activity and low physical activity is defined as unhealthful physical activity.

Healthful drinking status is defined as not currently consuming any alcohol and unhealthful drinking status is defined as currently consuming alcohol. Drinking more than 12 times in the last year is considered currently drinking (23).

In summary, the range of values for the lifestyle scores was 0–5. In addition, the lifestyle scores were divided into three groups for the analysis, where individuals with scores of 0 and 1 were classified as the unhealthful lifestyle group, those with scores of 2 and 3 were classified as the intermediate lifestyle group, and those with scores of 4 and 5 were classified as the healthful lifestyle group (30).

### Genetic Risk Score

Blood samples of subjects were collected and then used to detect SNPs by using a custom SNPscan^TM^ kit (Genesky Biotechnologies Inc., Shanghai, China) (31). SNP loci were screened based on the results of large GWAS studies reported in East Asia, and these loci were replicated in the study population. In total, all subjects were tested for 13 SNP loci associated with blood pressure levels (rs11191548, rs1275988, rs16849225, rs7136259, rs17249754, rs2107595, rs9810888, rs10745332, rs1378942, rs16998073, rs1902859, rs2021783, and rs7577262).

Based on these effect values, the SNPs with small effects were integrated into GRS to represent the genetic information. The genotype of each SNP locus was treated as a dummy variable, the effect value of each dummy variable was calculated in the study population (Supplementary Table 3), and the sum of the effect values of the genotypes of all SNP was summed to obtain the GRS. The GRS was then divided into low, intermediate, and high GRS risk groups, according to percentile.

### Epidemiological Indicators

To express the public health significance of adherence to the healthful lifestyle, the values of the epidemiologically relevant indicators [relative risk (RR), attributable risk (AR), population attributable risk (PAR), and prevented fraction for the population (PFP)] were calculated. The AR of an event is calculated as the number of people with the actual experience of the event divided by the total number of people exposed to the exposure factor, which is the likelihood that the event will occur in the risk population. The RR of an event is the likelihood of occurrence after exposure to an exposure factor, compared to the likelihood of occurrence in the non-exposed group. The PAR refers to the fraction of events in the risk population that are specifically attributable to the exposure factor, while PAR% is the percentage of events that could have been eliminated in the general population if the exposure factor had been eliminated (32). In the calculation of RR, AR, and PAR, lifestyle scores of 0, 1, 2, and 3 were regarded as an exposure factor, and lifestyle scores of 4 and 5 were regarded as a non-exposure factor.

Prevented fraction for the population is a complementary metric to PAR, which can be interpreted as a reduction in the disease burden or mortality due to existing exposure levels compared to the entire population without exposure (33). Since PFP calculates how much hypertension onset can be reversed based on the current prevalence of a healthful lifestyle, those with lifestyle scores of 4 and 5 were treated as the exposed group in the calculation.

### Statistical Analysis

All analyses were performed using SAS (version 9.1) and R (version 4.0.3). A significance level of 0.05 was used. Means ± SD and relative and absolute frequencies were used to express continuous and categorical variables, respectively. The *T*-test and chi-square test were used to compare the differences of continuous and categorical variables between hypertension and non-hypertension groups, respectively. The separate and joint associations of lifestyle factors and genetic factors with hypertension were performed using logistic regression, whereas the associations of lifestyle factors and genetic factors with blood pressure (including SBP and DBP) were performed using generalized linear models due to the short follow-up. The relative excess risk due to the interaction (RERI) was used to estimate additive interaction between lifestyle (healthful lifestyle vs. intermediate and unhealthful lifestyle) and GRS (upper 50th percentile vs. lower 50th percentile) for hypertension, SBP, and DBP. The RERI >0 suggests a positive interaction, RERI <0 suggests a negative interaction, and RERI = 0 suggests no interaction. In addition, the distribution and density of SBP and DBP in different groups are shown with violin plots. Age, sex, use of antihypertensive medicine, family history of hypertension, educational level, marriage, income, baseline SBP, and baseline DBP were used as covariates in conducting the analysis. We used 25 kg/m^2^, suggested as overweight by WHO, as the cut-off value for healthful and unhealthful BMI to generate lifestyle scores as a sensitivity analysis to verify the effect of different BMI cut-off values on the results.

## Results

### General Characteristics of the Study Population

Among the 4,592 subjects, 868 cases of hypertension onset (prevalence: 18.9%), of which the mean age was 49.04 year, with 36.93% of men. Table 1 shows the characteristics of hypertensive and non-hypertensive populations. Compared to the non-hypertensive population, the hypertensive population had older age, more men, higher educational level, less healthful BMI, higher GRS, more often had a family history of hypertension, and higher levels of baseline SBP and DBP. In addition, the distribution of the lifestyle score was different between the two populations. Also, Supplementary Table 4 demonstrates the distribution of different lifestyle factors in the GRS and lifestyle score subgroups.

**Table 1:** Characteristics of hypertensive and non-hypertensive populations.

	**Total (*n* = 4592)**	**Hypertension (*n* = 868)**	**Non-Hypertension (*n* = 3724)**	***P***
Age, mean ± sd	49.04 ± 11.52	53.59 ± 11.49	47.98 ± 11.27	<0.001
Men, *n* (%)	1,696 (36.93)	350 (40.32)	1,346 (36.14)	0.022
**Educational level**, ***n*****(%)**				<0.001
Primary and below	2,112 (45.99)	452 (52.07)	1,660 (44.58)	
Junior	1,962 (42.73)	322 (37.10)	1,640 (44.04)	
Senior and above	518 (11.28)	94 (10.83)	424 (11.39)	
Married/cohabit, n (%)	4,262 (92.85)	792 (91.45)	3,470 (93.18)	0.076
**Per capita monthly income**, ***n*****(%)**				0.736
<1,000 (RMB)	4,174 (91.10)	788 (91.20)	3,386 (91.07)	
1,000~ (RMB)	308 (6.72)	60 (6.94)	248 (6.67)	
3,000~ (RMB)	100 (2.18)	16 (1.85)	84 (2.26)	
**Diet**, ***n*****(%)**				0.064
Healthful diet	1,820 (39.63)	320 (36.87)	1,500 (40.28)	
Unhealthful diet	2,772 (60.37)	548 (63.13)	2,224 (59.72)	
**BMI**, ***n*****(%)**				<0.001
Healthful BMI	2,218 (48.30)	326 (37.56)	1,892 (50.81)	
Unhealthful BMI	2,374 (51.70)	542 (62.44)	1,832 (49.19)	
**Smoking**, ***n*****(%)**				0.214
Healthful smoking status	3,378 (73.56)	624 (71.89)	2,754 (73.95)	
Unhealthful smoking status	1,214 (26.44)	244 (28.11)	970 (26.05)	
**Physical activity**, ***n*****(%)**				0.614
Healthful physical activity	2,342 (51.00)	436 (50.23)	1,906 (51.18)	
Unhealthful physical activity	2,250 (49.00)	432 (49.77)	1,818 (48.82)	
**Drinking**, ***n*****(%)**				0.897
Healthful drinking status	4,004 (87.20)	758 (87.33)	3,246 (87.16)	
Unhealthful drinking status	588 (12.80)	110 (12.67)	478 (12.84)	
**Lifestyle score**, ***n*****(%)**				<0.001
0	50 (1.09)	12 (1.38)	38 (1.02)	
1	320 (6.97)	68 (7.83)	252 (6.77)	
2	1,132 (24.65)	242 (27.88)	890 (23.90)	
3	1,544 (33.62)	314 (36.18)	1,230 (33.03)	
4	1,184 (25.78)	190 (21.89)	994 (26.69)	
5	362 (7.88)	42 (4.84)	320 (8.59)	
GRS, mean ± sd	1.06 ± 0.41	1.16 ± 0.38	1.04 ± 0.41	<0.001
Family history of hypertension, *n* (%)	1,394 (30.36)	302 (34.79)	1,092 (29.32)	0.002
Baseline SBP, mean ± sd (mmHg)	116.06 ± 11.44	125.15 ± 9.20	113.94 ± 10.85	<0.001
Baseline DBP, mean ± sd (mmHg)	73.62 ± 7.59	78.70 ± 6.71	72.44 ± 7.29	<0.001

*BMI, body mass index; GRS, genetic risk score; SD, standard deviation*.

### Separate Association of Lifestyle and GRS With 3-Year Risk of Hypertension and Evaluated Blood Pressure

**Figure 1:**
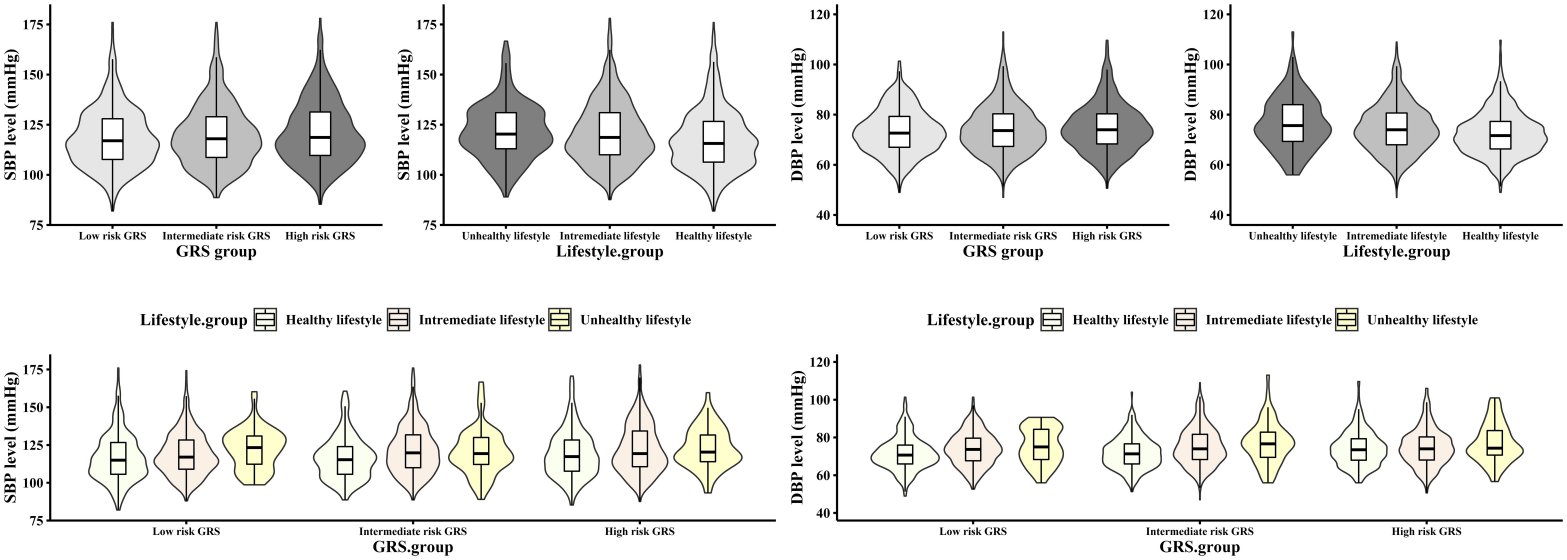
Distribution of SBP and DBP levels in genetic and lifestyle risk groups. The box plot shows the location of the quantile and the external shape shows the density at any location. DBP, diastolic blood pressure; GRS, genetic risk score; SBP, systolic blood pressure.

Table 2 shows the separate relationship of lifestyle and GRS with blood pressure and hypertension. Results showed that participants with a healthful lifestyle had an odds ratio (OR) (95% CI) of 0.774 (0.491, 1.221) than those with unhealthful lifestyle, and SBP and DBP levels were reduced by 1.234 and 1.463 mmHg, respectively. In addition, higher GRS was associated with a higher risk of having hypertension [OR and 95% CI: 1.878 (1.447, 2.437)] and elevated blood pressure. Furthermore, Figure 1 demonstrates the distribution of SBP and DBP levels in different GRS and lifestyle score subgroups. In the subgroups of GRS and lifestyle score, the levels of SBP and DBP increased with the increase of GRS risk and the decrease of lifestyle score. Further, Supplementary Table 5 demonstrates the separate association between individual lifestyle factors and hypertension, SBP, and DBP. After adjusting for covariates, GRS, and lifestyle factors other than themselves, unhealthful diet, BMI, smoking status, and drinking status are statistically significantly associated with hypertension and higher blood pressure levels.

**Table 2:** Association of lifestyle and genetic risk score (GRS) with 3-year risk of hypertension and evaluated blood pressure.

	**Hypertension** ** OR (95% CI)**	**SBP level** ** β (95% CI)**	**DBP level** ** β (95% CI)**
**Lifestyle**			
**Model 1**			
Unhealthful lifestyle	Reference	Reference	Reference
Intermediate lifestyle	0.951 (0.730, 1.239)	−0.726 (−2.419, 0.967)	−1.851 (−2.872, −0.830)
Healthful lifestyle	0.640 (0.482, 0.850)	−4.393 (−6.159, −2.627)	−3.916 (−4.981, −2.851)
**Model 2**			
Unhealthful lifestyle	Reference	Reference	Reference
Intermediate lifestyle	0.959 (0.640, 1.437)	0.101 (−1.334, 1.536)	−0.753 (−1.623, 0.116)
Healthful lifestyle	0.774 (0.491, 1.221)	−1.234 (−2.811, 0.343)	−1.463 (−2.419, −0.508)
**Model 3**			
Unhealthful lifestyle	Reference	Reference	Reference
Intermediate lifestyle	0.959 (0.640, 1.437)	0.101 (−1.334, 1.536)	−0.753 (−1.623, 0.1160)
Healthful lifestyle	0.774 (0.491, 1.221)	−1.234 (−2.811, 0.343)	−1.463 (−2.419, −0.508)
**GRS**			
**Model 1**			
Low risk GRS	Reference	Reference	Reference
Intermediate risk GRS	1.436 (1.184, 1.741)	1.451 (0.344, 2.559)	0.984 (0.314, 1.653)
High risk GRS	1.920 (1.593, 2.313)	2.959 (1.851, 4.067)	1.507 (0.837, 2.177)
**Model 2**			
Low risk GRS	Reference	Reference	Reference
Intermediate risk GRS	1.261 (0.962, 1.653)	0.445 (−0.436, 1.327)	0.361 (−0.173, 0.896)
High risk GRS	1.871 (1.443, 2.427)	1.381 (0.494, 2.268)	0.666 (0.128, 1.204)
**Model 3**			
Low risk GRS	Reference	Reference	Reference
Intermediate risk GRS	1.261 (0.961, 1.653)	0.423 (−0.458, 1.304)	0.343 (−0.191, 0.876)
High risk GRS	1.878 (1.447, 2.437)	1.395 (0.508, 2.281)	0.678 (0.140, 1.215)

*Low, intermediate, and high GRS risk were tertile 1, tertile 2, and tertile 3 of GRS, respectively; unhealthful, intermediate, and healthful lifestyle group were composed of LS for 0 and 1, LS for 2 and 3, and LS for 4 and 5, respectively. Hypertension, SBP level, and DBP level were the outcomes at the 3-year follow-up. Logistic regression was used to analyze the association of lifestyle and GRS with outcomes because of the short follow-up period. Model 1 was the crude model; Model 2 adjusted for age, sex, antihypertensive medicine, family history of hypertension, educational level, marriage, income, baseline SBP, and baseline DBP; Model 3 additionally adjusted for GRS (for an association of lifestyle with outcomes) or lifestyle score (for an association of GRS with outcomes). CI, confidence interval; GRS, genetic risk score; LS, lifestyle score*.

### Joint Effect of GRS and Lifestyle Score in Hypertension, SBP, and DBP

**Figure 2:**
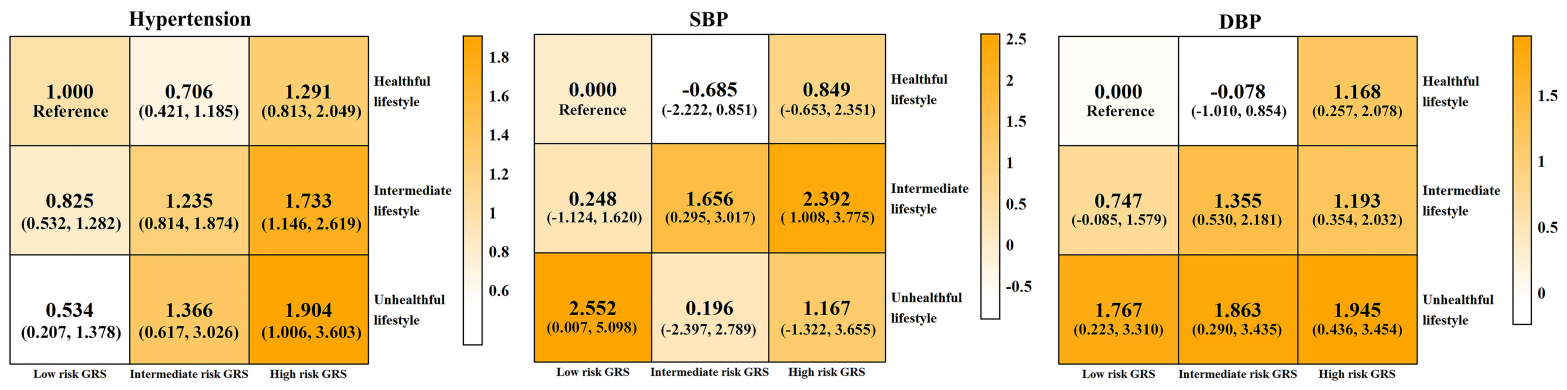
Combined effect of genetic and lifestyle on hypertension, SBP, and DBP. Low, intermediate, and high GRS risk were tertile 1, tertile 2, and tertile 3 of GRS, respectively; unhealthful, intermediate, and healthful lifestyle group were composed of LS for 0 and 1, LS for 2 and 3, and LS for 4 and 5, respectively. Hypertension, SBP level, and DBP level were the outcomes at the 3-year follow-up. Logistic regression was used to analyze the association of lifestyle and GRS with outcomes because of the short follow-up period. Take the participants with low risk of GSR and healthful lifestyle as the reference. The data in each square represent the effect size of each outcome [odds ratio (95% CI) for hypertension, β (95% CI) for SBP and DBP]. Covariates: age, sex, antihypertensive medicine, family history of hypertension, educational level, marriage, income, baseline SBP, and baseline DBP. The color bars on the right correspond to different effect values. No significant additive interaction effect of GRS and lifestyle score was observed (95% CI of RERI included 0). DBP, diastolic blood pressure; GRS, genetic risk score; SBP, systolic blood pressure.

The results in Figure 2 show the changes in risk of hypertension and the levels of SBP and DBP in the combined subgroups with GRS and lifestyle scores. In the figure, the darker the color of the square, the higher the risk of hypertension and the levels of blood pressure. With the unhealthier lifestyle and the higher risk of GRS, the darker the color of the square, that is, the higher the risk of hypertension and the higher level of blood pressure. In detail, using individuals with a healthful lifestyle in the low GRS risk group as a reference, the ORs (95% CI) for the risk of hypertension in the low, intermediate, and high GRS risk groups for individuals with the unhealthful lifestyle were 0.534 (0.207, 1.378), 1.366 (0.617, 3.026), and 1.904 (1.006, 3.603), respectively. Meanwhile, in the low, intermediate, and high GRS risk groups, the individuals with an unhealthful lifestyle had increased blood pressure levels of 2.552, 0.196, and 1.167 mmHg for SBP; 1.767, 1.863, and 1.945 mmHg for DBP compared to those with a healthful lifestyle. Individuals with a higher risk of GRS and a less healthful lifestyle had a higher risk of hypertension than those with a lower risk of GRS and a healthier lifestyle. With a higher risk of GRS and a less healthful lifestyle, the levels of SBP and DBP also increased. The detailed data are provided in Supplementary Table 6. In addition, Supplementary Table 7 shows RERI (95% CI) for hypertension, SBP, and DBP that are 0.212 (−0.326, 0.750), 7.047 (−1.855, 15.948), and 0.542 (−1.624, 2.708), respectively. No statistical evidence was observed for the additive interaction of genetic risk and lifestyle score with hypertension, SBP, or DBP.

Besides, Supplementary Figure 2 demonstrates the joint effect of different lifestyle factors with GRS risk groups on hypertension and SBP and DBP levels. The risk of an individual for hypertension and increased blood pressure increased with increase in unhealthful lifestyle factors and the GRS risk group.

### Epidemiological Indicators: RR, AR, PAR, and PFP

The epidemiological indicators in Table 3 suggest that the risk of developing hypertension in individuals with an unfavorable lifestyle (lifestyle score of 0–3) is 1.39 times higher than in individuals with a favorable lifestyle (lifestyle score of 4 or 5), and the increased incidence of hypertension due to an unfavorable lifestyle is 5.87%, while the incidence of hypertension due to an unfavorable lifestyle is 20.61% of the total population. The PFP values suggest that when the prevalence of favorable lifestyles in the population is at the current level, the incidence of hypertension in the total population can be reduced by 9.47%, reversing 90.80 hypertension cases, compared to the entire population with unfavorable lifestyles. Furthermore, at intermediate risk of GRS, adherence to a favorable lifestyle is more preventive (AR of 9.83 and PFP of 14.24%, reversing 48.16 hypertension onset).

**Table 3:** Epidemiological indicators of lifestyle on developing hypertension in different genetic risk subgroups.

	**Overall**	**Genetic risk subgroups**		
		**Low risk GRS**	**Intermediate risk GRS**	**High risk GRS**
No. of Events	868	214	290	364
Incidence rate, %	18.90	13.99	18.93	23.79
RR	1.39	1.13	1.80	1.34
AR, %	5.87	1.65	9.83	6.68
PAR, %	0.04	0.01	0.07	0.04
PAR%	20.61	7.84	35.31	18.10
PFP, %	9.47	3.79	14.24	9.08
Morbidities averted	90.80	8.44	48.16	36.34

*AR, attributable risk; PAR, population attributable risk; PAR%, population attributable risk proportion; PFP, prevented fraction for the population; RR, relative risk. RR, AR, PAR, and PAR% were calculated using the healthful lifestyle as the unexposed group and intermediate and unhealthful lifestyle as the exposed group, and the opposite was true when calculating PFP and morbidities averted*.

### Sensitivity Analysis

Supplementary Table 8 shows the results of sensitivity analysis. A lifestyle score was constructed using 25 as the cut-off value for healthful and unhealthful BMI, with other lifestyle factors held constant, as the sensitivity analysis. The results were found to be consistent with the main analysis results. Comparing to the individuals with healthful lifestyle in the low-risk GRS group, the individuals with unhealthful lifestyle in the high GRS group had an OR (95% CI) of 1.956 (1.004, 3.810) for hypertension, a β (95% CI) of 1.248 (−1.415, 3.911) for SBP, and a β (95% CI) of 1.887 (0.271, 3.503) for DBP. Thus, an unhealthful lifestyle leads to an increased risk of blood pressure, regardless of the genetic information.

## Discussion

This study explored the separate and joint effects of genetic factors and lifestyle on hypertension and blood pressure in a rural population. The findings suggested that GRS and lifestyle scores were associated with hypertension and blood pressure. Although there was a joint effect between genetic factors and lifestyle, there was no additive interaction effect between the two. Also, as the risk increased in the GRS and lifestyle score subgroups, the level of blood pressure increased with statistical significance in the intermediate and high GRS risk groups. In addition, the finding of PFP suggests that the healthful lifestyle could reverse hypertension onsets, especially, when the GRS risk is intermediate. Healthful lifestyle factors could reverse the genetic high risk of hypertension in the rural population, which can provide a clue for promoting a healthful lifestyle in the prevention and treatment of hypertension.

The association of genetic factors with blood pressure has been demonstrated in previous studies. Both SNP (14, 34, 35) and GRS (36, 37), integrating multiple SNP, were associated with blood pressure. In this study, we screened selected SNP from East Asian GWAS studies and replicated them in the rural Chinese population, the integration of the GRS using these SNP is highly correlated with hypertension and blood pressure. These results demonstrate that people carry genetic factors from birth that determine part of the risk of hypertension and high level of blood pressure.

The lifestyle score established in this study included five lifestyle factors, namely, diet, BMI, smoking, physical activity, and alcohol consumption. The risk of hypertension and level of blood pressure increased with the unhealthful lifestyle. A Sri Lankan study found an inverse relationship between a healthy lifestyle index (low BMI, inadequate physical activity, non-smoking, low alcohol intake, and adequate intake of fruits and vegetables) and hypertension (38). In addition, a study in the middle-aged and older Australian population has indicated that the higher the number of risk lifestyle factors, the greater the OR for hypertension (39). This study also found that a higher lifestyle score (i.e., healthier lifestyles) was associated with a lower risk of hypertension and lower blood pressure levels, suggesting that lifestyle can influence the risk of hypertension and blood pressure. In addition, a large number of studies have demonstrated that healthful lifestyles such as a healthful diet, lower BMI, no alcohol consumption, and adequate physical activity are associated with lower blood pressure levels (39–41). The results of the present study found similar findings, with healthful lifestyle factors being associated with a lower risk of hypertension and lower level of blood pressure (Supplementary Table 5). However, in the present population, smoking was not significantly associated with hypertension, which is the same conclusion reached in previous studies (42, 43). Therefore, the level of blood pressure decreases with a healthier lifestyle.

More importantly, this study found a joint effect between lifestyle and genetic factors in the risk of hypertension and blood pressure levels, meaning that the higher the genetic risk and the less healthful the lifestyle, the higher the tendency for blood pressure to rise, similar to the findings of previous studies (20, 44). An unhealthful lifestyle was associated with a higher risk of hypertension and higher levels of SBP and DBP in each GRS risk group, implying that a healthful lifestyle can compensate for the high level of blood pressure due to genetic factors. This was confirmed by the new epidemiological indicator PFP. In the total population, it would reverse 90 onsets of hypertension when all individuals adhered to 4 or more healthful lifestyles, compared to all individuals with <4 healthful lifestyles.

### Strengths and Limitations

The study has several noteworthy strengths: First, it is the first study to examine the separate and joint effects of genetic and lifestyle factors in hypertension and blood pressure in the resource-limited area. Second, this study not only statistically illustrates the association between lifestyle and hypertension but also explores the public health implications of lifestyle from a preventive medicine perspective using epidemiological indicators. However, several limitations deserve to be mentioned: first, data in the lifestyle scores were obtained from face-to-face questionnaires conducted by professionally trained interviewers, but most were self-reported, with the potential for over- or under-estimation, and, thus, the possibility of recall bias cannot be excluded. Furthermore, there is some degree of selection bias in the fact that the study included patients with hypertension who were taking antihypertensive medications and whose lifestyles had likely changed. Second, the definition of a healthful diet in this study used the CHEI, which uses the 3-day-24-h recalls method to collect information on diet, whereas our food information used the FFQ food collection scale. Although the methods used to collect dietary information are different, the scale we used can be used as a representative tool to evaluate the diet of the population in rural areas (45). We defined the diet of individuals with CHEI scores in the top 40% as a healthful diet and the bottom 60% as an unhealthful diet based on previous studies. However, our definition of a healthful diet was limited to our study population and there may be limitations to the extrapolation of the results. Third, the distribution of smoking among men and women in the population of this study is extremely uneven, which may lead to biased results. Fourth, because there is no standard or validated health effects of years of abstinence, the full complexity of smoking cessation may not be taken into account. In addition, the classification of lifestyles into high, intermediate, and low categories was not systematically validated, which requires rigorous validation by subsequent studies. Also, the findings of the study were applicable to rural populations, providing evidence for the health prevention of hypertension in rural populations. In addition, the lifestyle generation is based on the reported relevant literature and needs further validation as to whether it correctly reflects the lifestyles of individuals in this population, and thus to develop a precise and individualized hypertension control strategy in terms of a healthful lifestyle. In other words, it is important to address the definition of the specific healthful lifestyle and advocate for individuals to adapt their current lifestyle to make it healthy to prevent hypertension.

## Conclusion

This study elucidates the relationship between lifestyle and blood pressure in a rural Chinese population under different genetic risks, and also illustrates the significance of the healthful lifestyle in hypertension prevention. The results showed that the risk of hypertension increased with an unhealthful lifestyle under different risk genetic factors, but there was no significant interaction between the two. Besides, epidemiological indicators showed that compared to all individuals with intermediate and unhealthful lifestyles, it would reverse 90 hypertension onsets in the population due to the current prevalence of a healthful lifestyle. The findings of this study can draw public attention to a healthful lifestyle and may provide relevant evidence for the prevention and treatment of hypertension and individualized prevention strategies in rural populations.

## Data Availability Statement

The data are available from the corresponding author with reasonable justification.

## Ethics Statement

The studies involving human participants were reviewed and approved by Zhengzhou University Life Science Ethics Committee. The patients/participants provided their written informed consent to participate in this study.

## Author Contributions

CW and RB conceived and designed the experiments. MN, LZ, YW, RT, and XL gathered data. MN analyzed the data and drafted the manuscript. LZ and YW modified the manuscript. All authors contributed to the article and approved the submitted version.

## Conflict of Interest

The authors declare that the research was conducted in the absence of any commercial or financial relationships that could be construed as a potential conflict of interest.

## Publisher's Note

All claims expressed in this article are solely those of the authors and do not necessarily represent those of their affiliated organizations, or those of the publisher, the editors and the reviewers. Any product that may be evaluated in this article, or claim that may be made by its manufacturer, is not guaranteed or endorsed by the publisher.

## Abbreviations

GRS: genetic risk score
SNP: single nucleotide polymorphisms
BMI: body mass index
OR: odds ratio
CI: confidence interval
RR: relative risk
AR: attributable risk
PAR: population attributable risk
PFP: prevented fraction for the population
CVD: cardiovascular disease
GWAS: genome-wide association studies
SBP: systolic blood pressure
DBP: diastolic blood pressure
CHEI: Chinese healthy eating index
IPAQ: international physical activity questionnaire
WHO: world health organization
FFQ: food frequency questionnaire
RERI: relative excess risk due to interaction.
