# Supplementary material for: Lifestyle Score and Genetic Factors With Hypertension and Blood Pressure Among Adults in Rural China
Source: Front Public Health. 2021 Aug 17;9:687174. doi: 10.3389/fpubh.2021.687174 (PMC8416040; doi:10.3389/fpubh.2021.687174)
Supplement: Supplementary file 1 [file Table_1.DOCX]

**Table S1. Baseline characteristics of included and excluded participants**

|  | Excluded  (n=3676) | Included  (n=4592) | *P* |
| --- | --- | --- | --- |
| Age, mean±sd | 55.29±11.30 | 49.38±11.47 | <0.001 |
| Men, n (%) | 1234 (33.60) | 1696 (36.93) | 0.001 |
| Educational level, n (%) |  |  | <0.001 |
| Primary and below | 2230 (60.66) | 2112 (45.99) |  |
| Junior | 1196 (32.54) | 1962 (42.73) |  |
| Senior and above | 250 (6.80) | 518 (11.28) |  |
| Married/cohabit, n (%) | 3294 (89.71) | 4262 (92.85) | <0.001 |
| Per capita monthly income, n (%) |  |  | 0.562 |
| < 1000 (RMB) | 3354 (91.54) | 4174 (91.10) |  |
| 1000~ (RMB) | 242 (6.60) | 308 (6.72) |  |
| 3000~ (RMB) | 68 (1.86) | 100 (2.18) |  |
| Diet, n (%) |  |  | <0.001 |
| Healthful diet | 1318 (35.85) | 1820 (39.63) |  |
| Unhealthful diet | 2358 (64.15) | 2772 (60.37) |  |
| BMI, n (%) |  |  | <0.001 |
| Healthful diet | 1198 (32.59) | 2218 (48.30) |  |
| Unhealthful diet | 2478 (67.41) | 2374 (51.70) |  |
| Smoking, n (%) |  |  | <0.001 |
| Healthful smoking status | 2830 (76.99) | 3378 (73.56) |  |
| Unhealthful smoking status | 846 (23.01) | 1214 (26.44) |  |
| Drinking, n (%) |  |  | <0.001 |
| Healthful drinking status | 3392 (92.27) | 4004 (87.20) |  |
| Unhealthful drinking status | 284 (7.73) | 588 (12.80) |  |
| Physical activity, n (%) |  |  | <0.001 |
| Healthful physical activity | 2224 (60.50) | 2342 (51.00) |  |
| Unhealthful physical activity | 1452 (39.50) | 2250 (49.00) |  |
| Lifestyle score, mean±sd | 2.98±1.01 | 3.00±1.09 | 0.524 |
| Family history of hypertension, n (%) | 1322 (35.96) | 1394 (30.36) | <0.001 |
| Baseline SBP, mean±sd (mmHg) | 140.52±21.52 | 116.06±11.44 | <0.001 |
| Baseline DBP, mean±sd (mmHg) | 86.04±12.16 | 73.62±7.59 | <0.001 |

BMI: body mass index; sd: standard deviation. Differences of continuous variables between excluded and included were tested with Students t-test, Differences of categorical variables between excluded and included were tested with chi-square test.
