# Supplementary material for: Lifestyle Score and Genetic Factors With Hypertension and Blood Pressure Among Adults in Rural China
Source: Front Public Health. 2021 Aug 17;9:687174. doi: 10.3389/fpubh.2021.687174 (PMC8416040; doi:10.3389/fpubh.2021.687174)
Supplement: Supplementary file 2 [file Table_2.DOCX]

**Table S2. lifestyle factor definitions**

| **Lifestyle factor** | **Categories** | **Definitions** | **Score** |
| --- | --- | --- | --- |
| Diet | Healthful | Chinese Healthy Eating Index (CHEI) in the top 40 percentile (including the 40th percentile) | 1 |
|  | Unhealthful | CHEI in the bottom 60 percentile | 0 |
| BMI | Healthful | 18.5 ≤ BMI (kg/m2) ≤23.9 | 1 |
|  | Unhealthful | BMI < 18.5 & BMI > 23.9 | 0 |
| Smoking status | Healthful | Never smoke & Quit for 30 years or more | 1 |
|  | Unhealthful | Current smoke & Quit less than 30 years | 0 |
| Physical activity | Healthful | Moderate and high physical activity according to IPAQ | 1 |
|  | Unhealthful | Low physical activity according to IPAQ | 0 |
| Drinking status | Healthful | No current alcohol consumption | 1 |
|  | Unhealthful | Current alcohol consumption | 0 |

Current alcohol consumption defined as drinking more than or equal to 12 times in the past year.
