# Supplementary material for: Lifestyle Score and Genetic Factors With Hypertension and Blood Pressure Among Adults in Rural China
Source: Front Public Health. 2021 Aug 17;9:687174. doi: 10.3389/fpubh.2021.687174 (PMC8416040; doi:10.3389/fpubh.2021.687174)
Supplement: Supplementary file 3 [file Table_3.DOCX]

**Table S3. Single nucleotide polymorphisms used to build the GRS**

|  | **Risk allele** |  | **Effect size** | **OR (95% *CI*)** | ***P* value** |
| --- | --- | --- | --- | --- | --- |
| rs11191548 | **T** | C/C |  |  | 0.004 |
|  |  | C/T | 0.081 | 1.085 (0.791, 1.487) | 0.613 |
|  |  | T/T | 0.322 | 1.380 (1.014, 1.878) | 0.041 |
| rs1275988 | **C** | T/T |  |  | 0.001 |
|  |  | C/T | -0.044 | 0.957 (0.684, 1.340) | 0.799 |
|  |  | C/C | 0.266 | 1.305 (0.942, 1.806) | 0.109 |
| rs16849225 | **C** | T/T |  |  | 0.107 |
|  |  | C/T | -0.197 | 0.821 (0.672, 1.002) | 0.053 |
|  |  | C/C | -0.209 | 0.811 (0.654, 1.006) | 0.057 |
| rs7136259 | **C** | T/T |  |  | 0.016 |
|  |  | C/T | 0.317 | 1.373 (1.095, 1.722) | 0.006 |
|  |  | C/C | 0.315 | 1.371 (1.083, 1.734) | 0.009 |
| rs17249754 | **G** | A/A |  |  | 0.001 |
|  |  | G/A | 0.483 | 1.622 (1.262, 2.083) | <0.001 |
|  |  | G/G | 0.447 | 1.563 (1.210, 2.019) | 0.001 |
| rs2107595 | **A** | G/G |  |  | 0.558 |
|  |  | G/A | 0.085 | 1.089 (0.929, 1.276) | 0.293 |
|  |  | A/A | 0.076 | 1.079 (0.824, 1.413) | 0.578 |
| rs9810888 | **G** | T/T |  |  | 0.036 |
|  |  | G/T | 0.097 | 1.102 (0.928, 1.307) | 0.268 |
|  |  | G/G | 0.285 | 1.330 (1.071, 1.653) | 0.010 |
| rs10745332 | **A** | G/G |  |  | 0.014 |
|  |  | G/A | 0.293 | 1.340 (0.879, 2.044) | 0.174 |
|  |  | A/A | 0.060 | 1.062 (0.703, 1.604) | 0.776 |
| rs1378942 | **C** | A/A |  |  | 0.878 |
|  |  | C/A | -0.122 | 0.885 (0.551, 1.422) | 0.613 |
|  |  | C/C | -0.116 | 0.890 (0.562, 1.410) | 0.620 |
| rs16998073 | **T** | A/A |  |  | 0.077 |
|  |  | A/T | 0.192 | 1.212 (1.025, 1.433) | 0.024 |
|  |  | T/T | 0.141 | 1.151 (0.918, 1.444) | 0.223 |
| rs1902859 | **C** | T/T |  |  | 0.159 |
|  |  | C/T | 0.168 | 1.182 (0.996, 1.403) | 0.055 |
|  |  | C/C | 0.109 | 1.115 (0.894, 1.392) | 0.334 |
| rs2021783 | **C** | T/T |  |  | 0.819 |
|  |  | C/T | -0.117 | 0.890 (0.613, 1.292) | 0.539 |
|  |  | C/C | -0.090 | 0.914 (0.637, 1.312) | 0.626 |
| rs7577262 | **G** | A/A |  |  | 0.125 |
|  |  | G/A | -0.130 | 0.878 (0.693, 1.111) | 0.278 |
|  |  | G/G | 0.034 | 1.035 (0.817, 1.311) | 0.777 |

GRS: genetic risk score; OR: odds ratio; CI: confidence interval.
