# Supplementary material for: Lifestyle Score and Genetic Factors With Hypertension and Blood Pressure Among Adults in Rural China
Source: Front Public Health. 2021 Aug 17;9:687174. doi: 10.3389/fpubh.2021.687174 (PMC8416040; doi:10.3389/fpubh.2021.687174)
Supplement: Supplementary file 4 [file Table_4.DOCX]

| **Lifestyle score** | **Low GRS** | | | **Intermediate GRS** | | | **High GRS** | | |
| --- | --- | --- | --- | --- | --- | --- | --- | --- | --- |
|  | Healthful | Intermediate | Unhealthful | Healthful | Intermediate | Unhealthful | Healthful | Intermediate | Unhealthful |
| Healthful diet, % | 69.14 | 26.34 | 16.39 | 70.20 | 24.03 | 18.64 | 72.43 | 25.00 | 10.77 |
| Healthful BMI, % | 73.83 | 36.61 | 22.95 | 75.92 | 35.28 | 18.64 | 69.85 | 40.65 | 27.69 |
| Healthful smoking status, % | 96.88 | 68.75 | 6.56 | 94.69 | 71.65 | 6.78 | 95.96 | 69.39 | 6.15 |
| Healthful physical activity, % | 83.98 | 37.50 | 9.84 | 86.12 | 37.23 | 18.64 | 86.03 | 34.81 | 7.69 |
| Healthful drinking status, % | 98.44 | 89.51 | 31.15 | 99.18 | 87.88 | 18.64 | 97.79 | 88.55 | 38.46 |

**Table S4. Distribution of lifestyle risk factors per subgroups of genetic risk score and lifestyle score**

GRS: genetic risk score.
