# Supplementary material for: Lifestyle Score and Genetic Factors With Hypertension and Blood Pressure Among Adults in Rural China
Source: Front Public Health. 2021 Aug 17;9:687174. doi: 10.3389/fpubh.2021.687174 (PMC8416040; doi:10.3389/fpubh.2021.687174)
Supplement: Supplementary file 5 [file Table_5.DOCX]

**Table S5. Associations of individual lifestyle factors with hypertension and blood pressure**

| **Outcomes** |  | **Model 1** | ***P*** | **Model 2** | ***P*** | **Model 3** | ***P*** |
| --- | --- | --- | --- | --- | --- | --- | --- |
| Hypertension | Healthful diet | 0.866 (0.743, 1.009) | 0.064 | 0.832 (0.664, 1.042) | 0.109 | 0.829 (0.661, 1.039) | 0.104 |
|  | Healthful BMI | 0.582 (0.500, 0.678) | <0.001 | 0.808 (0.650, 1.004) | 0.054 | 0.793 (0.637, 0.987) | 0.037 |
|  | Healthful smoking status | 0.901 (0.764, 1.062) | 0.215 | 0.915 (0.637, 1.314) | 0.631 | 0.922 (0.641, 1.327) | 0.663 |
|  | Healthful physical activity | 0.963 (0.830, 1.116) | 0.614 | 0.895 (0.719, 1.115) | 0.324 | 0.918 (0.736, 1.145) | 0.448 |
|  | Healthful drinking status | 1.015 (0.813, 1.267) | 0.897 | 1.118 (0.769, 1.627) | 0.558 | 1.104 (0.759, 1.606) | 0.603 |
| SBP level | Healthful diet | -0.954 (-1.881, -0.028) | 0.044 | -0.692 (-1.452, 0.069) | 0.075 | -0.680 (-1.439, 0.079) | 0.079 |
|  | Healthful BMI | -3.249 (-4.151, -2.346) | <0.001 | -0.456 (-1.205, 0.294) | 0.234 | -0.490 (-1.239, 0.258) | 0.199 |
|  | Healthful smoking status | -2.391 (-3.417, -1.365) | <0.001 | 0.284 (-1.022, 1.589) | 0.670 | 0.301 (-1.002, 1.604) | 0.651 |
|  | Healthful physical activity | -0.281 (-1.188, 0.627) | 0.544 | -0.192 (-0.945, 0.560) | 0.616 | -0.180 (-0.931, 0.571) | 0.639 |
|  | Healthful drinking status | -2.477 (-3.832, -1.122) | <0.001 | -2.046 (-3.305, -0.787) | 0.001 | -2.057 (-3.314, -0.801) | 0.001 |
| DBP level | Healthful diet | 0.175 (-0.385, 0.735) | 0.540 | -0.073 (-0.532, 0.386) | 0.755 | -0.067 (-0.526, 0.391) | 0.773 |
|  | Healthful BMI | -3.685 (-4.222, -3.147) | <0.001 | -1.178 (-1.631, -0.725) | <0.001 | -1.195 (-1.647, -0.742) | <0.001 |
|  | Healthful smoking status | -0.950 (-1.570, -0.329) | 0.003 | -0.025 (-0.813, 0.764) | 0.951 | -0.016 (-0.804, 0.771) | 0.968 |
|  | Healthful physical activity | -0.036 (-0.584, 0.512) | 0.898 | 0.078 (-0.376, 0.533) | 0.735 | 0.084 (-0.370, 0.538) | 0.716 |
|  | Healthful drinking status | -3.003 (-3.818, -2.188) | <0.001 | -1.504 (-2.265, -0.744) | <0.001 | -1.510 (-2.270, -0.750) | <0.001 |

Model 1 was the crude model; model 2 adjusted for age, sex, antihypertensive medicine, family history of hypertension, educational level, marriage, income, baseline SBP, baseline DBP, and lifestyle factors other than itself; model 3 additionally adjusted for GRS. Hypertension, SBP level, and DBP level were the outcomes at the 3-year follow-up. Logistic regression was used to analyze the association of lifestyle and GRS with outcomes because of the short follow-up period. The effect value for hypertension is odds ratio, for SBP and DBP are *β*.
