# Supplementary material for: Lifestyle Score and Genetic Factors With Hypertension and Blood Pressure Among Adults in Rural China
Source: Front Public Health. 2021 Aug 17;9:687174. doi: 10.3389/fpubh.2021.687174 (PMC8416040; doi:10.3389/fpubh.2021.687174)
Supplement: Supplementary file 6 [file Table_6.DOCX]

**Table S6. Risk of Hypertension, SBP, and DBP in subgroup of genetic and lifestyle**

| Subgroup | No. of subjects | **Hypertension**  Adjusted *OR* (95% *CI*) | **SBP level**  Adjusted *β* (95% *CI*) | **DBP level**  Adjusted *β* (95% *CI*) |
| --- | --- | --- | --- | --- |
| **Low risk GRS** |  |  |  |  |
| Healthful lifestyle | 512 | Reference | Reference | Reference |
| Intermediate lifestyle | 896 | 0.825 (0.532, 1.282) | 0.248 (-1.124,1.620) | 0.747 (-0.085, 1.579) |
| Unhealthful lifestyle | 122 | 0.534 (0.207, 1.378) | 2.552 (0.007, 5.098) | 1.767 (0.223, 3.310) |
| **Intermediate risk GRS** |  |  |  |  |
| Healthful lifestyle | 490 | 0.706 (0.421, 1.185) | -0.685 (-2.222, 0.851) | -0.078 (-1.010, 0.854) |
| Intermediate lifestyle | 924 | 1.235 (0.814, 1.874) | 1.656 (0.295, 3.017) | 1.355 (0.530, 2.181) |
| Unhealthful lifestyle | 118 | 1.366 (0.617, 3.026) | 0.196 (-2.397, 2.789) | 1.863 (0.290, 3.435) |
| **High risk GRS** |  |  |  |  |
| Healthful lifestyle | 544 | 1.291 (0.813, 2.049) | 0.849 (-0.653, 2.351) | 1.168 (0.257, 2.078) |
| Intermediate lifestyle | 856 | 1.733 (1.146, 2.619) | 2.392 (1.008, 3.775) | 1.193 (0.354, 2.032) |
| Unhealthful lifestyle | 130 | 1.904 (1.006, 3.603) | 1.167 (-1.322, 3.655) | 1.945 (0.436, 3.454) |

Low, Intermediate, and High GRS risk were tertile 1, tertile 2, and tertile 3 of GRS, respectively; Unhealthful, Intermediate, and Healthful lifestyle group were composed of LS for 0 and 1, LS for 2 and 3, and LS for 4 and 5, respectively. Hypertension, SBP level, and DBP level were the outcomes at the 3-year follow-up. Logistic regression was used to analyze the association of lifestyle and GRS with outcomes because of the short follow-up period. Covariates: age, sex, antihypertensive medicine, family history of hypertension, educational level, marriage, income, baseline SBP, and baseline DBP. GRS: genetic risk score; SBP: systolic blood pressure; DBP: diastolic blood pressure; *OR*: odds ratio; *CI*: confidence interval.
