# Supplementary material for: Lifestyle Score and Genetic Factors With Hypertension and Blood Pressure Among Adults in Rural China
Source: Front Public Health. 2021 Aug 17;9:687174. doi: 10.3389/fpubh.2021.687174 (PMC8416040; doi:10.3389/fpubh.2021.687174)
Supplement: Supplementary file 7 [file Table_7.DOCX]

**Table S7.** Additive interaction between lifestyle (healthful lifestyle vs. intermediate and unhealthful lifestyle) and GRS (upper 50th percentile vs. lower 50th percentile) for hypertension, SBP, and DBP

|  | Hypertension | SBP level | DBP level |
| --- | --- | --- | --- |
| RERI | 0.212 (-0.326, 0.750) | 7.047 (-1.855, 15.948) | 0.542 (-1.624, 2.708) |

Covariates: age, sex, antihypertensive medicine, family history of hypertension, educational level, marriage, income, baseline SBP, and baseline DBP. The additive interaction is considered significant if the confidence interval does not contain the 0, and is positive interaction when RERI > 0, negative interaction otherwise. RERI: relative excess risk due to interaction.
