# Supplementary material for: Lifestyle Score and Genetic Factors With Hypertension and Blood Pressure Among Adults in Rural China
Source: Front Public Health. 2021 Aug 17;9:687174. doi: 10.3389/fpubh.2021.687174 (PMC8416040; doi:10.3389/fpubh.2021.687174)
Supplement: Supplementary file 8 [file Table_8.DOCX]

**Table S8. Risk of Hypertension, SBP, and DBP in subgroup of GRS and lifestyle (BMI >25kg/m^2^ defined as unhealthful BMI)**

| Subgroup | No. of subjects | **Hypertension**  Adjusted *OR* (95% *CI*) | **SBP level**  Adjusted *β* (95% *CI*) | **DBP level**  Adjusted *β* (95% *CI*) |
| --- | --- | --- | --- | --- |
| **Low risk GRS** |  |  |  |  |
| Healthful lifestyle | 574 | Reference | Reference | Reference |
| Intermediate lifestyle | 856 | 0.704 (0.459, 1.080) | 0.409 (-0.931, 1.748) | 0.649 (-0.164, 1.462) |
| Unhealthful lifestyle | 100 | 0.787 (0.309, 2.002) | 3.871 (1.155, 6.587) | 1.901 (0.253, 3.549) |
| **Intermediate risk GRS** |  |  |  |  |
| Healthful lifestyle | 566 | 0.626 (0.387, 1.011) | -0.368 (-1.809, 1.073) | 0.068 (-0.807, 0.943) |
| Intermediate lifestyle | 866 | 1.264 (0.852, 1.876) | 1.866 (0.535, 3.198) | 1.315 (0.507, 2.123) |
| Unhealthful lifestyle | 100 | 1.181 (0.503, 2.771) | 0.058 (-2.682, 2.797) | 1.151 (-0.512, 2.813) |
| **High risk GRS** |  |  |  |  |
| Healthful lifestyle | 590 | 1.218 (0.788, 1.882) | 1.006 (-0.426, 2.438) | 1.209 (0.340, 2.078) |
| Intermediate lifestyle | 834 | 1.639 (1.111, 2.420) | 2.556 (1.210, 3.901) | 1.026 (0.209, 1.843) |
| Unhealthful lifestyle | 106 | 1.956 (1.004, 3.810) | 1.248 (-1.415, 3.911) | 1.887 (0.271, 3.503) |

Define BMI >25kg/m^2^ as unhealthy and recalculate lifestyle scores for sensitivity analysis. Low, Intermediate, and High GRS risk were tertile 1, tertile 2, and tertile 3 of GRS, respectively; Unhealthful, Intermediate, and Healthful lifestyle group were composed of LS for 0 and 1, LS for 2 and 3, and LS for 4 and 5, respectively. Hypertension, SBP level, and DBP level were the outcomes at the 3-year follow-up. Logistic regression was used to analyze the association of lifestyle and GRS with outcomes because of the short follow-up period. Co-variables: age, sex, antihypertensive medicine, family history of hypertension, educational level, marriage, income, baseline SBP, and baseline DBP. GRS: genetic risk score; SBP: systolic blood pressure; DBP: diastolic blood pressure; *OR*: odds ratio; *CI*: confidence interval.
