# Supplementary figures and images for: Lifestyle Score and Genetic Factors With Hypertension and Blood Pressure Among Adults in Rural China
Source: Front Public Health. 2021 Aug 17;9:687174. doi: 10.3389/fpubh.2021.687174 (PMC8416040; doi:10.3389/fpubh.2021.687174)

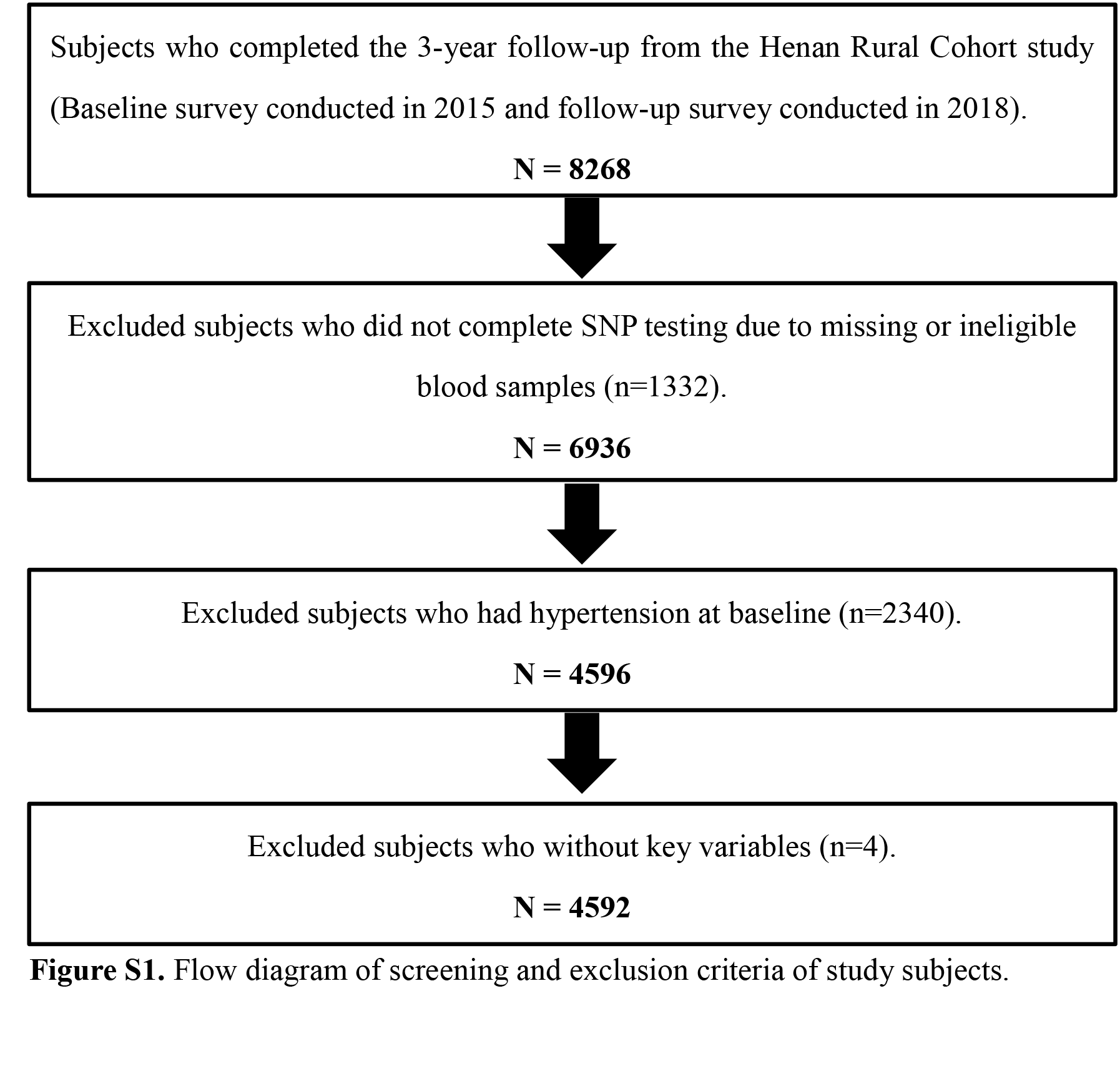

Supplement: Supplementary file 9 [file Image_1.TIF]

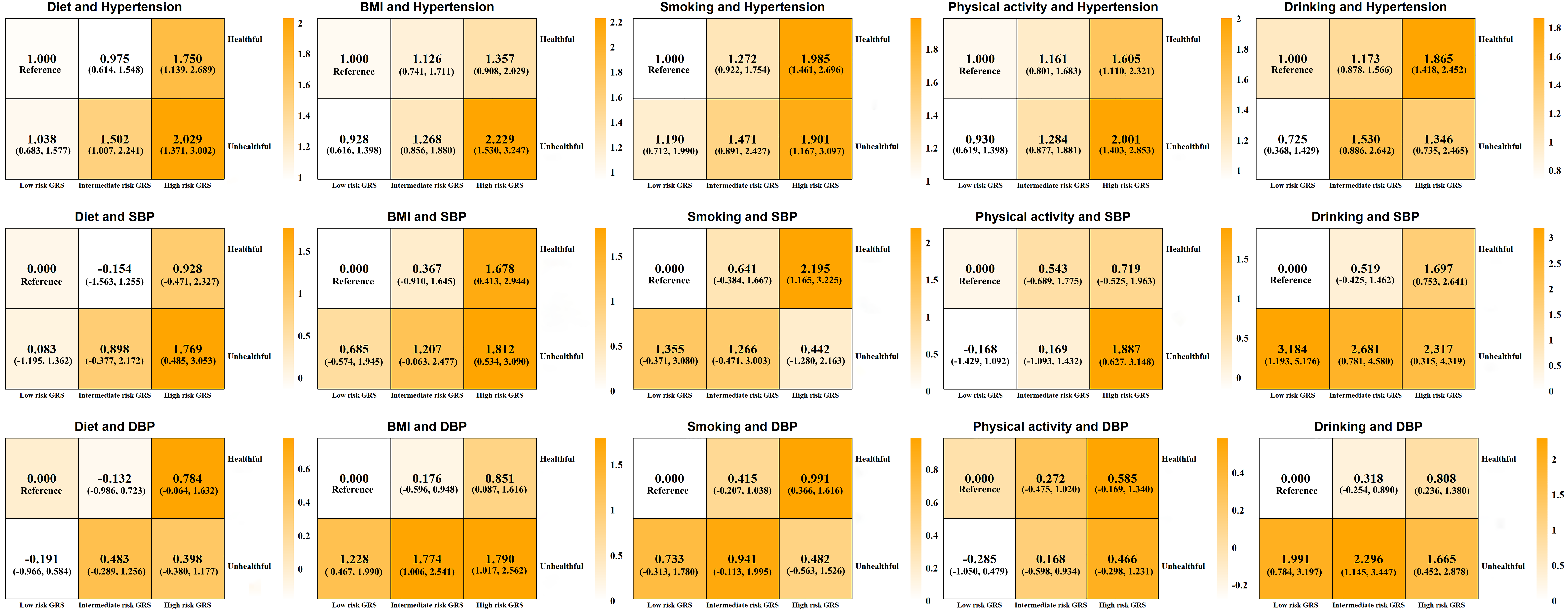

Supplement: Supplementary file 10 [file Image_2.TIF]
